# The Prescription trends and dosing appropriateness analysis of novel oral anticoagulants in ischemic stroke patients: a retrospective study of 9 cities in China

**DOI:** 10.3389/fphar.2024.1304139

**Published:** 2024-03-12

**Authors:** Mingfen Wu, Hailun Jiang, Kefu Yu, Zhigang Zhao, Bin Zhu

**Affiliations:** Department of Pharmacy, Beijing Tiantan Hospital, Capital Medical University, Beijing, China

**Keywords:** novel oral anticoagulants, NOACs, ischemic stroke, prescription trends, dosing appropriateness

## Abstract

**Background:** Novel oral anticoagulants (NOACs) have been recommended by guidelines as the first-line drugs for preventing cardiogenic stroke. We aimed to provide an overview of the prescription trends and dosing appropriateness of NOACs in China.

**Methods:** We conducted a retrospective analysis of NOAC prescriptions using the Hospital Prescription Analysis Cooperation Project data from 2016 to 2022. Various patient features, such as gender, age, city, year, source, department visited, original diagnosis, dosing, cost, and insurance type, were collected and analyzed to examine the trends and dosing appropriateness of NOAC usage in ischemic stroke patients.

**Results:** 62,014 NOAC prescriptions were analyzed, including 16,602 for dabigatran, 45,253 for rivaroxaban, and 159 for apixaban. 85.14% of the patients were aged 65 or above, and tertiary hospitals accounted for 95.97% of NOAC prescriptions. NOAC prescriptions rose from 1828 in 2016 to 13,998 in 2021 but dropped to 13,166 in 2022. The percentage of annual prescriptions for NOACs among stroke patients has increased from 0.05% in 2016 to 0.37% in 2022. Total drug cost increased from ¥704541.18 in 2016 to ¥4128648.44 in 2021, then decreased to ¥1680109.14 in 2022. Prescriptions were divided into 48,321 appropriate and 11,262 inappropriate dosing groups, showing significant differences in medications, age, year, city type, hospital level, source, insurance type, and department visited (all *p* < 0.001). The median drug cost for inappropriate dosing was higher than for appropriate dosing (¥55.20 VS ¥83.80). The top comorbidities in ischemic stroke patients were atrial fibrillation (35.30%), hypertension (32.75%), and coronary heart disease (16.48%).

**Conclusion:** The application of NOACs in the Chinese population is increasing. Our findings highlight the frequent deviation from labeled dosing of NOACs in clinical practice. Continued efforts are necessary to promote the appropriate use of NOACs according to the standard dosage in the drug insert.

## Introduction

Ischemic stroke is a leading cause of neurological morbidity and mortality in neurological diseases worldwide (GBD, 2017 Disease and Injury Incidence and Prevalence Collaborators, 2018). Recent epidemiological data reveals that in 2019, there were approximately 12.2 million incident cases of stroke and 101 million prevalent cases of stroke worldwide. Among these cases, ischemic stroke accounted for 62.4% of all incident strokes (GBD, 2019 Stroke Collaborators, 2021). Several factors can lead to the development of ischemic stroke, including arteriosclerosis affecting cerebral circulation, occlusion of small and medium-sized cerebral blood vessels, and embolism originating from the heart (cardiac embolism). Among the various causes of ischemic stroke, cardioembolic causes have been found to contribute to an increasing proportion of cases, and this trend is expected to continue in the coming decades (Kamel and Healey, 2017). Compared to non-cardiac stroke, atrial fibrillation (AF) related stroke exhibits a more unfavorable clinical outcome, with higher rates of recurrence, disability, and mortality (Yiin et al., 2019).

Anticoagulation therapy is considered to be the cornerstone of ischemic stroke prevention in non-valvular atrial fibrillation (NVAF) patients. Both vitamin K antagonists (VKAs) and novel oral anticoagulants (NOACs) have been proven effective in preventing stroke in patients with NVAF, ultimately prolonging life. NOACs have demonstrated superior efficacy and safety compared to VKAs(Ruff et al., 2014; Nazha et al., 2018; Tsai et al., 2020; Li et al., 2021), and they also have advantages such as rapid onset, short half-life, minimal drug interactions, and the absence of regular monitoring of the international normalized ratio (INR). They are extensively utilized for stroke prevention in AF patients, effectively reducing the formation of thrombosis and lowering the risk of recurrent stroke. Therefore, the current guidelines from Europe, North America, Japan, and China strongly advocate for NOACs as the first-line treatment for AF patients with a prior history of cardioembolic stroke, recommending lifelong anticoagulation therapy for secondary stroke prevention, unless contraindications are present (Kleindorfer et al., 2021; Steffel et al., 2021; Gladstone et al., 2022; Miyamoto et al., 2022; Liping et al., 2023). Furthermore, NOACs are particularly suitable for Asian patients, as they carry a significantly lower risk of bleeding and intracranial hemorrhage (ICH) while maintaining their effectiveness (Cho et al., 2019; Lee et al., 2020). NOACs have emerged as effective alternatives to warfarin for globally preventing cardiogenic stroke. Four specific NOACs, namely dabigatran, rivaroxaban, apixaban, and edoxaban, have obtained regulatory approval and gained guideline recommendations for secondary prevention of stroke in AF patients (Carnicelli et al., 2022). NOACs do not require dose adjustments based on clinical monitoring indicators, as the standard dose is clearly stated in the package insert and guidelines. However, it is common in clinical practice to prescribe NOACs at dosages that deviate from the recommended standard dosage in the drug insert. Nevertheless, there is currently limited understanding of the prescription patterns and dosing appropriateness of NOACs in Chinese patients with ischemic stroke in real-world settings. Therefore, this study aims to provide a comprehensive overview of the prescription trends and dosing appropriateness of NOACs for ischemic stroke in China.

## Materials and methods

### Data sources

This study was a retrospective analysis based on real-world prescription data. The data was obtained from the Hospital Prescription Analysis Cooperative Project (HPACP) database, which was conducted by the Chinese Pharmaceutical Association. The HPACP database included prescriptions from 120 medical institutions in 9 cities across China, including Beijing, Chengdu, Guangzhou, Harbin, Hangzhou, Shanghai, Shenyang, Tianjin, and Zhengzhou. A random sampling method was used to select 10 workday prescriptions for each quarter in the HPACP database, excluding national statutory holidays. Various patient features, such as gender, age, region, year, hospital level, source, department visited, original diagnosis, drug name, dosage, frequency, cost, and insurance type, were collected and analyzed to investigate the trends and dosing appropriateness of NOAC usage in ischemic stroke patients. The personal information of patients and clinicians was anonymized in the extracted prescriptions. The study received approval from the Ethics Committee of Beijing Tiantan Hospital, Capital Medical University.

### Sample extraction

The population we studied was patients who were using NOACs for anticoagulation therapy to prevent secondary stroke following an ischemic stroke. In the present study, prescriptions meeting the following criteria were included: 1) prescribed for patients diagnosed with ischemic stroke, with diagnostic keywords including stroke, cerebral apoplexy, ischemic cerebrovascular disease, cerebral infarction, transient ischemic attack, cerebral embolism, and cerebral thrombosis; 2) encompassing outpatient, emergency, and inpatient patients; 3) containing at least one of the following NOACs: Dabigatran, Rivaroxaban, Apixaban, Edoxaban; and 4) prescribed between the years 2016 and 2022. Prescriptions with missing age or sex information were excluded.

### Statistical analysis

In this study, we conducted a descriptive analysis of the total number, proportion, cost, and trends of prescriptions. The median and interquartile range (IQR) were used to describe continuous variables that did not follow a normal distribution. Categorical variables were presented as frequency (%). The prescriptions were divided into two groups: appropriate dosing and inappropriate dosing, and the baseline characteristics of the two groups were analyzed using chi-square tests and nonparametric tests. *p* < 0.05 was considered statistically significant. All data were analyzed using Microsoft Excel and IBM SPSS Statistics (version 25; IBM Corporation, United States).

## Results

### Demographic features of the studied population

**FIGURE 1:**
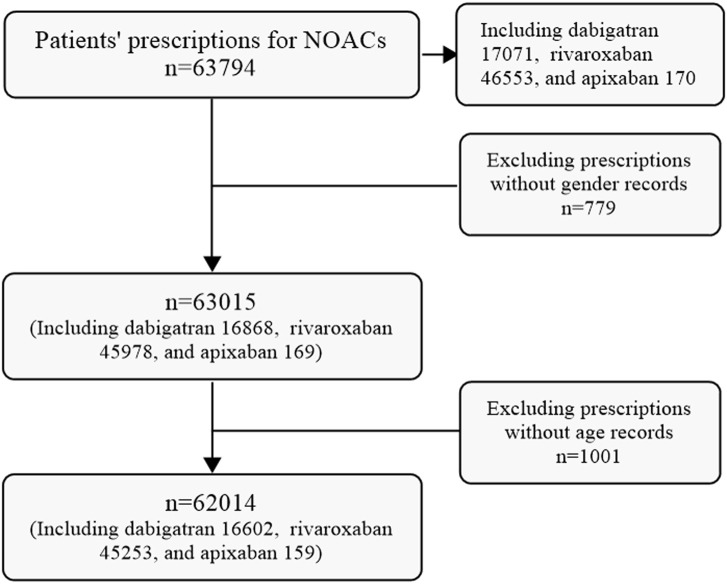
Flow chart of prescription screening in this study.

A total of 63,794 prescriptions for NOACs were initially extracted from the CPDHP database. Following refinement and screening processes, 62,014 prescriptions including dabigatran, Edoxaban and Apixaban, were selected for analysis in this study. While no prescriptions for edoxaban were identified among the extracted data (Figure 1).

The demographic features of the studied population are shown in Table 1. Most patients with ischemic stroke who used NOACs were 65 years or above, comprising 85.14% of the study population. Tertiary hospitals accounted for 95.70% of NOAC prescriptions, with 66.08% of these patients being from inpatient wards. Among the 9 cities included in the study, First-tier cities Beijing, Shanghai, and Guangzhou contributed to approximately half of the total prescriptions (Supplementary Table S1). 75.54% of patients using NOACs received full or partial reimbursement from Medicare.

**TABLE 1:** Demographic features of the studied population prescribed for NOACs.

Characteristic	Ⅱa inhibitors	Xa inhibitors		Total NOACs
	Dabigatran	Rivaroxaban	Apixaban	
Gender, n (%)				
Male	9560 (57.58)	24924 (55.08)	74 (46.50)	34558 (55.73)
Female	7042 (42.42)	20329 (44.92)	85 (53.50)	27456 (44.27)
Age, [median (IQR)]^*^	76 (67–83)	79 (70–87)	76 (70–85)	78 (69–86)
<18	1 (0.00)	6 (0.01)	0	7 (0.01)
18–64	2947 (17.75)	6242 (13.79)	20 (12.58)	9209 (14.85)
≥65	13654 (82.24)	39005 (86.19)	139 (87.42)	52798 (85.14)
City type, n (%)				
First-tier	9112 (54.88)	22563 (49.86)	81 (50.94)	31756 (51.21)
Other	7490 (45.12)	22690 (50.14)	78 (49.06)	30258 (48.79)
Hospital-Level, n (%)				
Tertiary hospitals	16109 (97.03)	43081 (95.20)	159 (100)	59349 (95.70)
Primary or secondary	493 (2.97)	2172 (4.80)	0	2665 (4.30)
Patient source, n (%)				
Outpatient	7214 (43.45)	13120 (29.00)	70 (44.03)	20404 (32.90)
Emergency	98 (0.59)	533 (1.17)	1 (0.63)	632 (1.02)
Inpatient	9290 (55.96)	31600 (69.83)	88 (55.34)	40978 (66.08)
Insurance Type, n (%)				
Full or partial	12341 (74.33)	34426 (76.07))	79 (49.69)	46846 (75.54)
Self-pay	2764 (16.65)	4653 (10.28)	33 (20.75)	7450 (12.01)
Others	1497 (9.02)	6174 (13.65)	47 (29.56)	7718 (12.45)
Department Visited, n (%)				
Neurology	7785 (46.89)	15982 (35.32)	55 (34.59)	23822 (38.41)
Cardiology	3994 (24.06)	6246 (13.80)	39 (24.53)	10279 (16.58)
Geriatrics	1888 (11.37)	9736 (21.51)	20 (12.58)	11644 (18.78)
Others	2935 (17.68)	13289 (29.37)	45 (28.30)	16269 (26.23)
^#^Cost [median (IQR)]^*^	171.80 (34.36–516.00)	51.30 (27.60–276.00)	54.88 (7.08–104.44)	55.20 (27.60–344.40)

*IQR, interquartile range, it is shown as (p25, p75).

^#^The cost is measured in Chinese yuan.

### Overall trends in the utilization of NOACs in ischemic stroke patients

**FIGURE 2:**
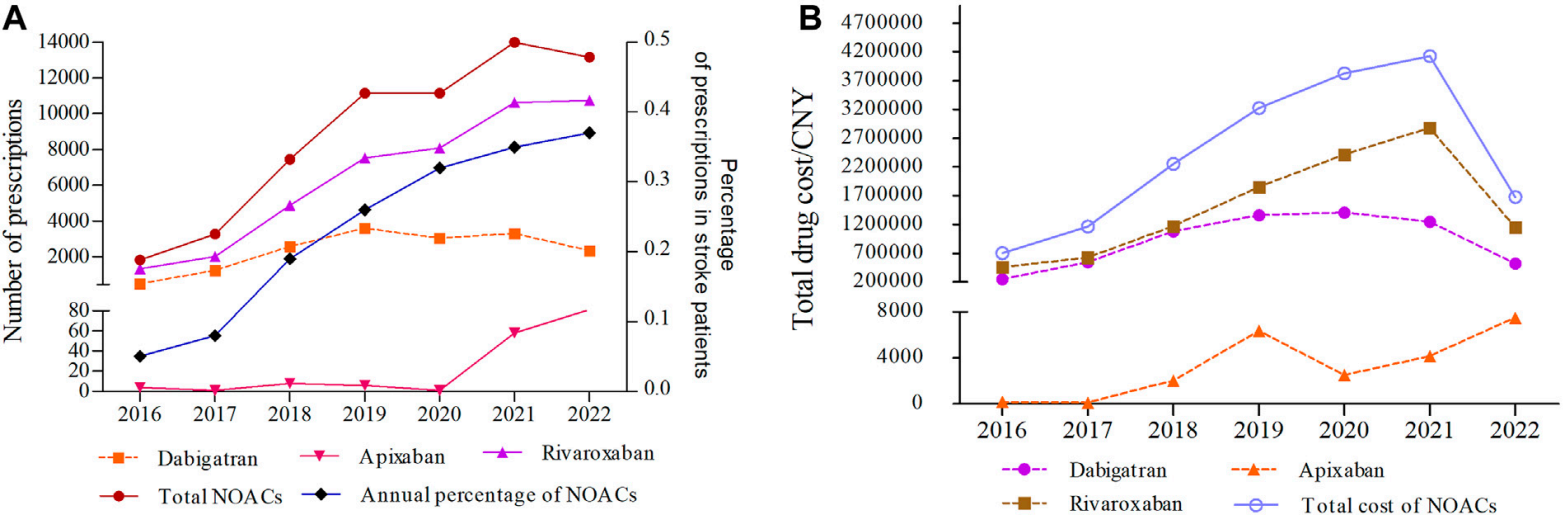
The annual trends in the number/percentage of prescriptions **(A)** and total drug cost for NOACs **(B)** from 2016 to 2022 (CNY, Chinese Yuan).

Figure 2 illustrates the annual trends in the number/percentage of prescriptions and total drug cost for NOACs from 2016 to 2022. During this period, the total number of prescriptions for NOACs experienced consistent growth, rising from 1828 in 2016 to 13,998 in 2021, marking a substantial increase of 6.66-fold. However, in 2022, the number of prescriptions slightly decreased to 13,166, showing a decline of 5.94% compared to the previous year, which was mainly attributed to the decline in dabigatran prescriptions in 2022. Notably, the percentage of annual prescriptions for NOACs among stroke patients continued to grow, from 0.05% in 2016 to 0.37% in 2022, suggesting that stroke patients who require anticoagulant therapy are increasingly choosing to use NOACs. The annual number and percentage of prescriptions for rivaroxaban demonstrated a significant surge, increasing approximately 8 times from 2016 to 2022. In contrast, apixaban had relatively few prescriptions, with only sporadic prescriptions between 2016 and 2020. Nonetheless, it exhibited a clear growth trajectory in the past 2 years (Figure 2A; Supplementary Table S2).

The annual trends in the total drug cost of NOACs from 2016 to 2022 are shown in Figure 2B. The total drug cost of NOACs experienced a substantial increase from ¥704541.18 in 2016 to ¥4128648.44 in 2021, rising by 4.86 times. However, in 2022, there was a rapid decline of 59.31% compared to the previous year. From 2016 to 2021, both dabigatran and rivaroxaban showed a consistent upward trajectory in terms of total drug cost, with dabigatran experiencing a 5.63-fold increase and rivaroxaban witnessing a 6.34-fold increase. These increments were in line with the corresponding rise in the number of prescriptions. However, a notable shift occurred in 2022, marked by a significant decline in the total drug cost (Figure 2B; Supplementary Tables S3, S4). The total drug costs for apixaban followed a similar pattern to the number of prescriptions, increasing steadily and continuing to rise from 2020 to 2022 (Figure 2B; Supplementary Table S5).

### Dosing appropriateness analysis of using NOACs

The prescriptions of NOACs were divided into appropriate dosing group and inappropriate dosing group according to the recommended standard dosage as stated in the drug insert. Taking into consideration the specific comorbidities and physical conditions of individual patients, we conducted a comprehensive assessment to determine the appropriate dosing range for NOACs. We defined the appropriate dosing range as those falling within the maximum and minimum dosages that align with the patient’s particular health conditions. Any prescriptions surpassing the maximum daily dosage or falling below the minimum effective dosage for the specific indications were classified as inappropriate dosing prescriptions. Conversely, prescriptions falling within the recommended dosing range specified in the drug insert were considered appropriate dosing prescriptions. More specifically, prescriptions were considered inappropriate when the daily dose of dabigatran exceeded 300 mg or fell below 150mg, rivaroxaban exceeded 30 mg or fell below 10 mg, and apixaban exceeded 20 mg or fell below 2.5 mg. Prescriptions with unclear dosages were excluded from the analysis, resulting in a total of 48,321 appropriate dosing prescriptions and 11,262 inappropriate ones.

The features of appropriate and inappropriate dosing prescriptions for NOACs are presented in Table 2. When comparing the baseline characteristics of the two groups, it was found that the median age was 78 years in the appropriate group and 80 years in the inappropriate group. In the inappropriate dosing group, more patients aged 65 years or above were prescribed NOACs than those aged 18–64 years (16.84% VS 2.06%), and more prescriptions were prescribed in other cities than in first-tier cities (10.44% VS 8.46%). Compared with the appropriate dosing group, the median drug cost of the inappropriate dosing group was higher (¥55.20 VS ¥83.80). There were significant differences between the two groups in terms of medications, age, year, city type, hospital level, patient source, insurance type, and department visited (all p˂0.001). However, there was no statistically significant difference in gender and total cost (*p* > 0.05).

**TABLE 2:** Comparison of the features of patients with appropriate and inappropriate dosing prescriptions for NOACs.

Features	Appropriate dosing	Inappropriate dosing	*p*-value
Total number, n (%)	48321 (81.10)	11262 (18.90)	
Medications, n (%)			<0.001
Dabigatran	14116 (23.69)	1966 (3.30)	
Rivaroxaban	34076 (57.19)	9274 (15.56)	
Apixaban	129 (0.22)	22 (0.04)	
Gender, n (%)			0.849
Male	27009 (45.33)	6306 (10.58)	
Female	21312 (35.77)	4956 (8.32)	
Age [median, (IQR)]^*^	78 (69–86)	80 (71–87)	<0.001
Age group, n (%)			<0.001
18–64	7589 (12.74)	1226 (2.06)	
≥65	40732 (68.36)	10036 (16.84)	
Year, n (%)			<0.001
2016	1569 (2.63)	186 (0.31)	
2017	2745 (4.61)	382 (0.64)	
2018	6103 (10.24)	1064 (1.79)	
2019	8894 (14.93)	1851 (3.11)	
2020	8547 (14.34)	2251 (3.78)	
2021	10588 (17.77)	2913 (4.89)	
2022	9875 (16.57)	2615 (4.39)	
City type, n (%)			<0.001
First-tier	25245 (42.37)	5043 (8.46)	
Other	23076 (38.73)	6219 (10.44)	
Hospital-Level, n (%)			<0.001
Tertiary hospitals	46738 (78.44)	10687 (17.94)	
Primary or secondary	1583 (2.66)	575 (0.97)	
Patient source, n (%)			<0.001
Outpatient	15510 (26.03)	4609 (7.74)	
Emergency	486 (0.82)	129 (0.22)	
Inpatient	32325 (54.25)	6524 (10.95)	
Insurance type, n (%)			<0.001
Full or partial	36769 (61.71)	8251 (13.85)	
Self-pay	6299 (10.57)	1012 (1.70)	
Others	5253 (8.82)	1999 (3.35)	
Department visited, n (%)			<0.001
Neurology	19166 (32.17)	3559 (5.97)	
Cardiology	7278 (12.21)	2773 (4.65)	
Geriatrics	8650 (14.52)	2698 (4.53)	
Others	13227 (22.20)	2232 (3.75)	
^#^Total cost, [median (IQR)]^*^	55.20 (27.60–343.80)	83.80 (27.60–414.00)	0.997

*IQR, interquartile range, it is shown as (p25, p75).

^#^Total cost is measured in Chinese yuan. Chi-square tests were used to compare categorical variables, and nonparametric tests were used to compare age and total cost.

**FIGURE 3:**
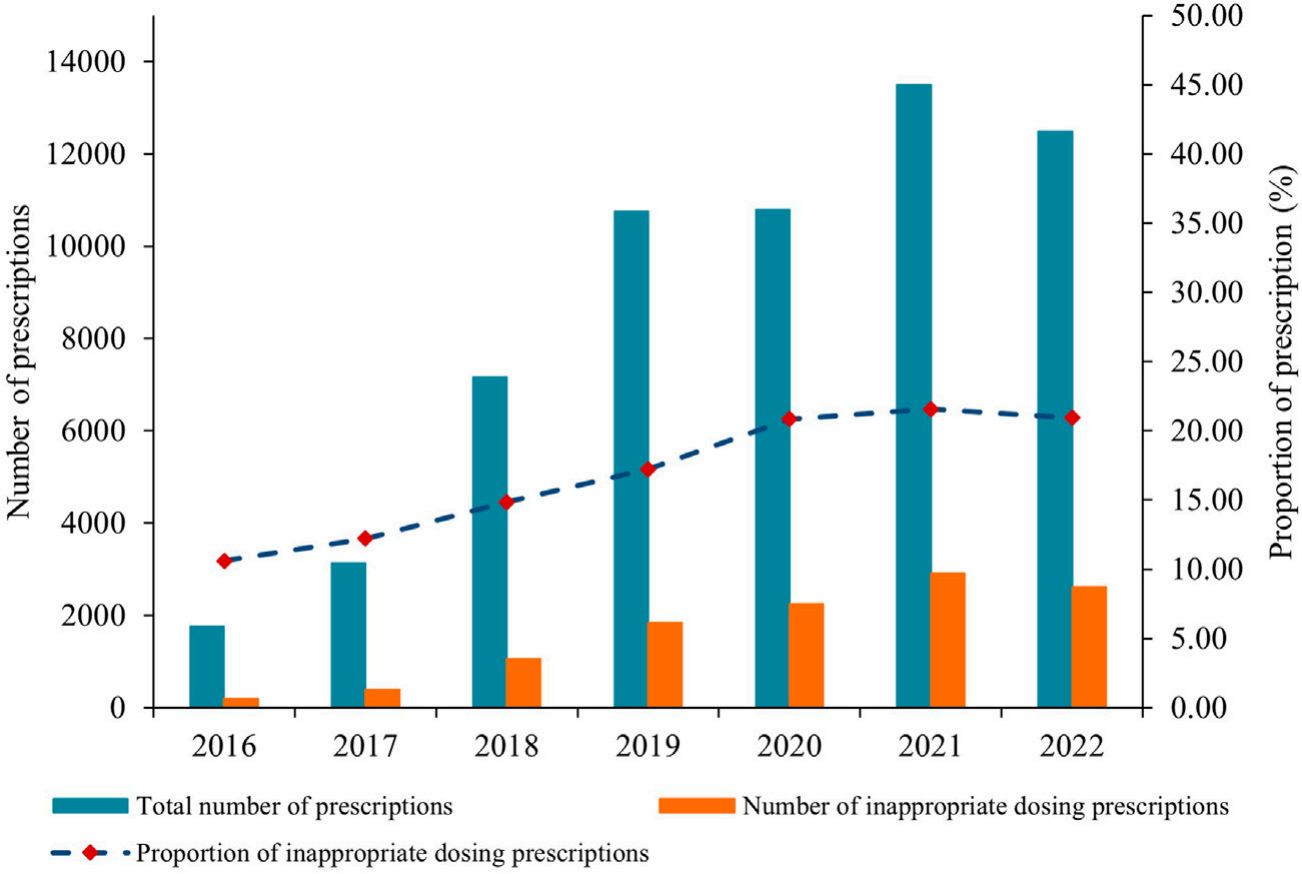
The number and proportion of inappropriate dosing prescriptions from 2016 to 2022.

The number of inappropriate dosing prescriptions increased in tandem with the total number of annual prescriptions, peaking in 2021 (Figure 3; Supplementary Table S6). Tertiary hospitals accounted for over 95% of these prescriptions, but their share has been gradually declining from 99.89% in 2016 to 94.69% in 2022. Conversely, prescriptions for NOACs in primary or secondary hospitals have remained relatively low, but they have shown consistent growth, rising from 2 in 2016 to 663 in 2022. Additionally, the number of appropriate dosing prescriptions in primary or secondary hospitals has also steadily increased alongside the total prescriptions (Supplementary Table S7).

**FIGURE 4:**
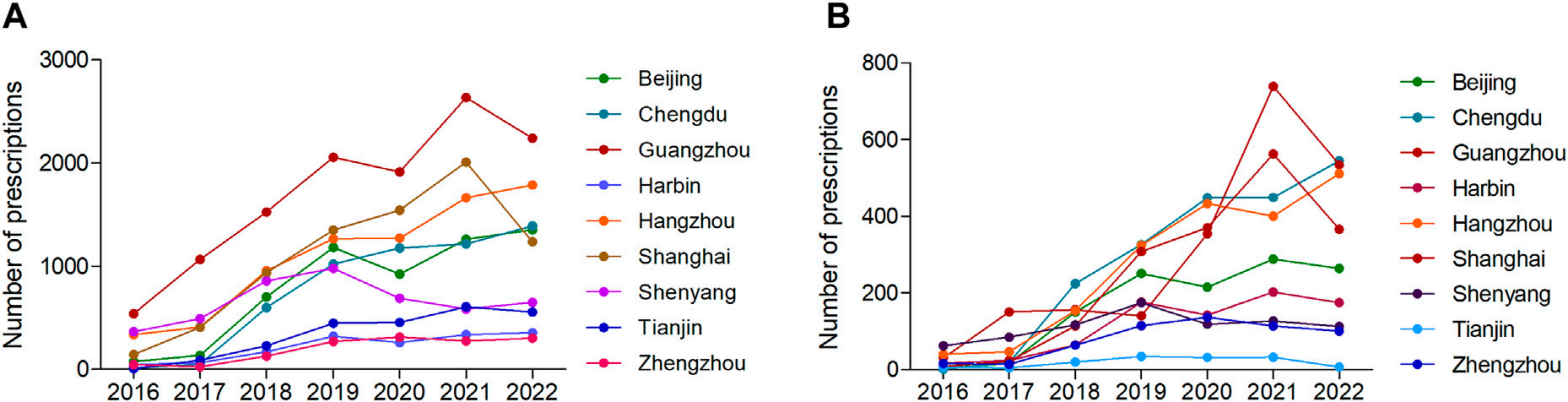
The number of **(A)** appropriate dosing prescriptions and **(B)** inappropriate dosing prescriptions in different cities from 2016 to 2022.

From the perspective of the prescription trend in different cities, the number of appropriate dosing prescriptions in Chengdu and Hangzhou showed consistent and continuous growth, while the other 7 cities showed corresponding decreases from 2020 to 2022**.** The annual number of appropriate dosing prescriptions in Guangzhou has been ranked first among the 9 cities (Figure 4A; Supplementary Table S8). The number of inappropriate dosing prescriptions in Chengdu and Hangzhou had been increasing continuously, while the other 7 cities showed a significant decline in 2022 (Figure 4B; Supplementary Table S9).

**FIGURE 5:**
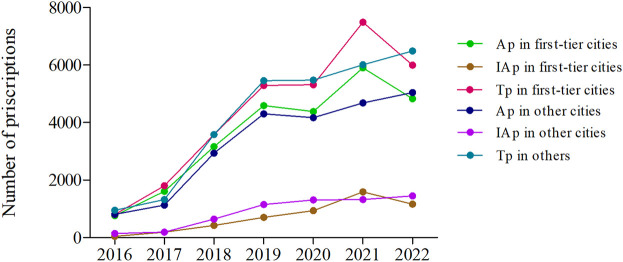
The annual trends of appropriate and inappropriate dosing prescriptions in different types of cities from 2016 to 2022. (Ap, appropriate dosing prescriptions; IAp, inappropriate dosing prescriptions; Tp, total prescriptions; First-tier cities include Beijing, Guangzhou, and Shanghai. Other cities include Chengdu, Harbin, Hangzhou, Shenyang, Tianjin, and Zhengzhou.)

The annual trends of appropriate and inappropriate dosing prescriptions in different types of cities are presented in Figure 5. In the first-tier cities, the appropriate dosing and total prescriptions showed two declining inflection points in 2020 and 2022 respectively. In 2022, the appropriate dosing prescriptions and total prescriptions decreased by 26.70% and 19.97%, respectively. In other cities, the appropriate dosing and total prescriptions only decreased in 2020 and continued to increase from 2021 to 2022. For another, the number of inappropriate dosing prescriptions decreased in 2022 in the first-tier cities, while in other cities has been growing slowly from 2016 to 2022 (Figure 5; Supplementary Table S10).

### Analysis of comorbidities

To determine the prevalence of comorbidities in patients with ischemic stroke who received NOAC treatment, we conducted a comprehensive analysis based on the diagnostic information provided in the prescriptions. Among the patients who used NOACs for ischemic stroke, a significant majority (88.94%) had cerebral infarction. AF emerged as the most common comorbidity, accounting for 35.30% of cases, followed by hypertension (32.75%), coronary heart disease (16.48%), atherosclerosis (15.46%), diabetes mellitus (13.41%), cardiac insufficiency (10.08%), and hyperlipidemia (8.93%) (Table 3; Supplementary Figure S1).

**TABLE 3:** The top 20 comorbidities in patients with ischemic stroke using NOACs.

Rank	Diagnosis	Frequency	Proportion (%)	Rank	Diagnosis	Frequency	Proportion (%)
1	Cerebral Infarction	56736	88.94	11	Pulmonary Infection	3885	6.09
2	Atrial Fibrillation	22522	35.30	12	Osteoporosis	3076	4.82
3	Hypertension	20894	32.75	13	Benign Prostatic Hyperplasia	2688	4.21
4	Coronary Heart Disease	10516	16.48	14	Venous Thromboembolism	3691	5.79
5	Atherosclerosis	9864	15.46	15	Stenocardia	2463	3.86
6	Diabetes Mellitus	8553	13.41	16	Thyroid Disorder	2285	3.58
7	Cardiac Insufficiency	6432	10.08	17	Heart Failure	2062	3.23
8	Hyperlipidemia	5697	8.93	18	Cerebral Embolism	1983	3.11
9	Ischemic Cerebrovascular Disease	4483	7.03	19	Sleep Disorders	1912	3.00
10	Arrhythmia	4200	6.58	20	Anxiety-Depression	1442	2.26

## Discussion

This study investigated the utilization of NOACs in Chinese patients with ischemic stroke, using a large prescription database. The NOACs examined in the present study included dabigatran, rivaroxaban, and apixaban, while edoxaban was not detected. Considering the fluctuations in the number of stroke cases, we examined the percentage of NOAC prescriptions among stroke patients. Our analysis revealed a consistent annual rise in the proportion of NOAC prescriptions, indicating a growing preference among stroke patients in need of anticoagulant therapy for using NOACs. The growing preference for NOACs is attributed to their user-friendly nature, high effectiveness, safety profile, and expanding clinical evidence.

In terms of prescription frequency, rivaroxaban was the most prescribed, followed by dabigatran, with apixaban being the least. This discrepancy may be attributed to the time of NOACs covered under medical insurance and essential medicine. Rivaroxaban, a direct factor Xa inhibitor, was first approved for clinical use in China in 2009. It was subsequently included in the “National Reimbursement Drug List” (NRDL) that same year and was further incorporated into the “National Essential Drug List” (NEDL) in 2018 (National Health Commission of the People’s Republic of China, 2018). Rivaroxaban has thus garnered the most extensive safety experience among NOACs, which accounts for its highest number of prescriptions. Although both dabigatran and apixaban were approved for clinical use in 2013 and included in the NRDL in 2017 (Department of Human Resources and Social Security, 2017), dabigatran was additionally included in the NEDL in 2018 (National Health Commission of the People’s Republic of China, 2018), whereas apixaban was not. This disparity might explain the lower prescription proportion for apixaban. Edoxaban, approved for clinical use in late 2018, was included in the NRDL at the end of 2020 but has not yet been incorporated into the NEDL (Department of Human Resources and Social Security, 2020).

Our findings showed a steady increase in the frequency of NOAC use from 2016 to 2021 but decreased in 2022, which was mainly due to a decrease in the number of stroke patient visits in hospitals in 2022. Moreover, we observed a dramatic reduction in the total drug cost of NOACs in 2022, with a decline of 59.31% compared to 2021. This reduction can be attributed to the inclusion of dabigatran and rivaroxaban in the fifth batch of the National Drug Centralized Procurement List (NDCPL) in 2021, leading to a substantial reduction in drug prices. For instance, the price of the original drug rivaroxaban decreased by 49.95%, and the highest price of generic rivaroxaban decreased by 99.42%. Consequently, the implementation of the fifth batch of NDCPL in medical institutions nationwide in 2022 resulted in a quick and strong reduction in the total cost of NOACs.

The introduction of four NOACs with varying indications, doses, and criteria for dose reduction has made the process of determining the appropriate dosing more complex. This complexity poses a significant challenge in daily clinical practice and individualized treatment. The recommended dosing of NOACs for preventing stroke and systemic embolism in patients with AF in the Chinese and US guidelines is consistent with the label requirements (Steffel et al., 2018; Chinese Medical Association, 2021).

In the present study, we found that 18.90% of prescriptions for NOACs were either overdosing or underdosing, indicating that off-label dosing of NOACs in Chinese patients with ischemic stroke is common and needs more attention. Notably, a substantial proportion, approximately 40%, of AF patients receive NOAC dosing inconsistent with drug labeling in real-world clinical settings (Yu et al., 2020). These inconsistent prescribing patterns may be associated with poor safety and lack of efficacy benefits in patients with severe kidney disease. In patients with normal or mildly impaired renal function, these prescribing patterns may be associated with lower efficacy and no safety benefits (Yao et al., 2017). It is worth noting that overdosing on NOACs has been associated with worse clinical outcomes in Asian AF patients (Yu et al., 2020), suggesting that we should be more vigilant against overdosing use of NOACs in Chinese patients.

NOACs for the prevention of stroke in AF patients require dose adjustment based on certain clinical criteria (e.g., kidney function), but the off-label dosing of use is common. A recent study revealed that 12.6% of patients with NVAF did not adhere to the US Food and Drug Administration (FDA) label recommendations for NOAC administration, the incidence was higher in patients with worse renal function (31.9%) and was associated with less consistent long-term anticoagulation (Rymer et al., 2023). A Previous study showed that among the 1,473 patients with a renal indication for dose reduction, 43.0% were potentially overdosing, which was associated with a higher risk of major bleeding. Among 13,392 patients with no renal indication for dose reduction, 13.3% were potentially underdosing with NOACs, which was associated with a higher risk for stroke (Yao et al., 2017). Despite the potential benefits of lower doses of NOACs in patients with impaired renal function, those who receive less than the minimum recommended dosing still face an increased risk of thrombosis and poorer clinical outcomes. The AFIRE trial showed that underdose rivaroxaban (10 mg/day), compared with the standard dose (15 mg/day), was associated with a similar incidence of thrombotic events but a lower rate of bleeding events in AF patients with stable coronary artery disease (CAD) and preserved renal function (Arashi et al., 2022). A recent study revealed that utilization of lower-dose rather than standard-dose NOACs was associated with a higher incidence of death, stroke, and systemic embolism in patients with reduced kidney function (Harrington et al., 2023). These findings suggest that label adherence to NOAC dosing should be emphasized to achieve optimal clinical outcomes in Asian patients with AF.

Standard-dose anticoagulation is associated with a relatively good prevention of stroke and other embolism. One study showed that standard-dose apixaban (5 mg twice daily) was associated with significantly lower risks for stroke/systemic embolism and death than low-dose apixaban (2.5 mg twice daily) (Siontis et al., 2018). A network meta-analysis has shown that standard dose NOACs offer the most favorable risk-benefit profile compared to other oral anticoagulants. In Asian patients, standard-dose NOACs are a more attractive treatment option than underdosing NOACs due to their significant reduction in ischemic stroke without an increased risk of major bleeding (Cha et al., 2017). However, with safety concerns about increasing bleeding, off-label underdosing of NOACs is common in East Asian patients with AF (Cho et al., 2019; Chan et al., 2020). A study of oral anticoagulant dose adjustment in patients with acute ischemic stroke related to AF showed that one-third of patients with ischemic stroke used an inappropriate dosing regimen during oral Xa inhibitor therapy (26% underdose and 8% overdose), and underdosing was associated with lower functional plasma levels, higher clinical stroke severity, and worse functional outcome (Stoll et al., 2020). In another study involving Asian patients with NVAF, the risks of ischemic stroke, all-cause death, and composite clinical outcomes were higher in the off-label underdosing apixaban group than the on-label standard dose apixaban group, but the risks of major bleeding were comparable (Lee et al., 2021). These findings highlight the need for efforts to improve the quality and dosing of NOACs. We discovered a consistent increase in the number of inappropriate dosing prescriptions in Chengdu and Hangzhou from 2016 to 2022. This finding highlights the need for further investigation into the prescribing practices of physicians in these two cities. Identifying the factors influencing prescribed dosing, implementing standardized training and promotion, and advocating for the rational use of NOACs are essential steps to address this issue.

Our study also has some limitations. Firstly, this study only analyzed NOACs and did not include information on other anticoagulants, which limits our ability to investigate the overall changes in anticoagulation treatment for cardiogenic stroke. Secondly, the database did not provide information on treatment efficacy and adverse effects, preventing us from determining the effectiveness and safety of NOACs in patients. Thirdly, trends in the number and proportion of NOAC prescriptions have limitations in capturing the full picture of drug consumption. Lastly, this study mainly focused on samples from developed large cities in China and therefore may not accurately represent the situation in less developed areas and rural regions.

## Conclusion

The application of NOACs in the Chinese population is increasing. The total cost of NOACs in ischemic stroke has been steadily increasing since 2016 but showed a sharp decline in 2022, demonstrating that the “drug price negotiation policy” in China have significantly contributed to the reduction in drug costs. Atrial fibrillation, hypertension, coronary heart disease, atherosclerosis, and diabetes were the most prevalent comorbidities among patients with ischemic stroke. In addition, our findings highlight the frequent deviation from labeled dosing of NOACs in clinical practice, which may result in poor efficacy in thromboprophylaxis and pose a high safety risk. Thus, it is crucial to improve adherence to the standard dosages of NOACs to enhance the effectiveness of stroke prevention. Continued efforts are necessary to promote the appropriate use of NOACs according to the standard dosage in the drug insert.

## Data Availability

The original contributions presented in the study are included in the article/Supplementary Material, further inquiries can be directed to the corresponding authors.
