# Supplementary material for: The Prescription trends and dosing appropriateness analysis of novel oral anticoagulants in ischemic stroke patients: a retrospective study of 9 cities in China
Source: Front Pharmacol. 2024 Mar 12;15:1304139. doi: 10.3389/fphar.2024.1304139 (PMC10963614; doi:10.3389/fphar.2024.1304139)
Supplement: Supplementary file 1 [file Table1.DOCX]

**Table S1.** Distribution of prescriptions of NOACs in different cities

| **City** | **Number of prescriptions, n (%)** | | | **Total NOACs** |
| --- | --- | --- | --- | --- |
|  | **Dabigatran** | **Rivaroxaban** | **Apixaban** |  |
| Beijing* | 2948 (17.76) | 4382 (9.68) | 0 (0.00) | 7330 (11.82) |
| Shenyang | 1465 (8.82) | 4295 (9.49) | 2 (1.26) | 5769 (9.30) |
| Chengdu | 1719 (10.35) | 5735 (12.67) | 50 (31.45) | 7454 (12.02) |
| Guangzhou* | 3808 (22.94) | 10998 (24.30) | 64 (40.25) | 14808 (23.88) |
| Harbin | 627 (3.78) | 2149 (4.75) | 0 (0.00) | 2778 (4.48) |
| Hangzhou | 2911 (17.53) | 6694 (14.79) | 17 (10.69) | 9655 (15.57) |
| Shanghai* | 2356 (14.19) | 7183 (15.87) | 17 (10.69) | 9556 (15.41) |
| Tianjin | 108 (0.65) | 2429 (5.37) | 2 (1.26) | 2554 (4.12) |
| Zhengzhou | 660 (3.98) | 1388 (3.07) | 9 (5.66) | 2062 (3.33) |
| Total | 16602(100) | 45253 (100) | 159 (100) | 62014 (100) |

Note: NOACs, novel oral anticoagulants; * represents the first-tier city.
