# Supplementary material for: The Prescription trends and dosing appropriateness analysis of novel oral anticoagulants in ischemic stroke patients: a retrospective study of 9 cities in China
Source: Front Pharmacol. 2024 Mar 12;15:1304139. doi: 10.3389/fphar.2024.1304139 (PMC10963614; doi:10.3389/fphar.2024.1304139)
Supplement: Supplementary file 2 [file Table8.docx]

**Table S8.** The number of appropriate dosing prescriptions for NOACs in different regions from 2016 to 2022.

| City  Year | Beijing | Chengdu | Guangzhou | Harbin | Hangzhou | Shanghai | Shenyang | Tianjin | Zhengzhou | Total |
| --- | --- | --- | --- | --- | --- | --- | --- | --- | --- | --- |
| 2016 | 77 | 12 | 540 | 45 | 335 | 144 | 366 | 5 | 45 | 1569 |
| 2017 | 137 | 51 | 1067 | 64 | 411 | 409 | 493 | 88 | 25 | 2745 |
| 2018 | 701 | 599 | 1527 | 172 | 955 | 937 | 856 | 228 | 128 | 6103 |
| 2019 | 1181 | 1021 | 2056 | 322 | 1266 | 1351 | 978 | 448 | 271 | 8894 |
| 2020 | 923 | 1176 | 1913 | 261 | 1270 | 1544 | 689 | 457 | 314 | 8547 |
| 2021 | 1262 | 1215 | 2635 | 337 | 1664 | 2008 | 585 | 605 | 277 | 10588 |
| 2022 | 1354 | 1391 | 2241 | 356 | 1786 | 1238 | 649 | 557 | 303 | 9875 |
| Total | 5635 | 5465 | 11979 | 1557 | 7687 | 7631 | 4616 | 2388 | 1363 | 48321 |
