# Supplementary material for: The Prescription trends and dosing appropriateness analysis of novel oral anticoagulants in ischemic stroke patients: a retrospective study of 9 cities in China
Source: Front Pharmacol. 2024 Mar 12;15:1304139. doi: 10.3389/fphar.2024.1304139 (PMC10963614; doi:10.3389/fphar.2024.1304139)
Supplement: Supplementary file 3 [file Table7.docx]

**Table S7.** The annual number of prescriptions for NOACs in hospitals of different levels from 2016 to 2022.

| Year | Prescriptions in tertiary hospitals | | | Prescriptions in primary or secondary hospitals | | |
| --- | --- | --- | --- | --- | --- | --- |
|  | Total number of prescriptions | Appropriate dosing prescriptions, n (%) | Inappropriate dosing prescriptions, n (%) | Total number of prescriptions | Appropriate dosing prescriptions, n (%) | Inappropriate dosing prescriptions, n (%) |
| 2016 | 1753 (99.89) | 1568 (89.45) | 185 (10.55) | 2 (0.11) | 1 (50.00) | 1 (50.00) |
| 2017 | 3047 (97.44) | 2665 (87.46) | 382 (12.54) | 80 (2.56) | 80 (100.00) | 0 (0.00) |
| 2018 | 7007 (97.77) | 5957 (85.01) | 1050 (14.99) | 160 (2.23) | 146 (91.25) | 14 (8.75) |
| 2019 | 10415 (96.93) | 8664 (83.19 | 1751 (16.81) | 330 (3.07) | 230 (69.70) | 100 (30.30) |
| 2020 | 10446 (96.74) | 8283 (79.29) | 2163 (20.71) | 352 (3.26) | 264 (75.00) | 88 (25.00) |
| 2021 | 12930 (95.77) | 10182 (78.75) | 2748 (21.25) | 571 (4.23) | 406 (71.10) | 165 (28.90) |
| 2022 | 11827 (94.69) | 9419 (79.64) | 2408 (20.36) | 663 (5.31) | 456 (68.78) | 207 (31.22) |
