# Supplementary material for: The Prescription trends and dosing appropriateness analysis of novel oral anticoagulants in ischemic stroke patients: a retrospective study of 9 cities in China
Source: Front Pharmacol. 2024 Mar 12;15:1304139. doi: 10.3389/fphar.2024.1304139 (PMC10963614; doi:10.3389/fphar.2024.1304139)
Supplement: Supplementary file 5 [file Table9.docx]

**Table S9.** The number of inappropriate dosing prescriptions for NOACs in different regions from 2016 to 2022.

| City  Year | Beijing | Chengdu | Guangzhou | Harbin | Hangzhou | Shanghai | Shenyang | Tianjin | Zhengzhou | Total |
| --- | --- | --- | --- | --- | --- | --- | --- | --- | --- | --- |
| 2016 | 4 | 2 | 31 | 17 | 40 | 8 | 62 | 6 | 16 | 186 |
| 2017 | 16 | 18 | 151 | 24 | 47 | 22 | 85 | 5 | 14 | 382 |
| 2018 | 150 | 224 | 156 | 65 | 154 | 114 | 117 | 20 | 64 | 1064 |
| 2019 | 251 | 326 | 141 | 176 | 324 | 308 | 175 | 35 | 115 | 1851 |
| 2020 | 215 | 448 | 354 | 143 | 433 | 370 | 119 | 32 | 137 | 2251 |
| 2021 | 288 | 449 | 738 | 202 | 400 | 562 | 127 | 33 | 114 | 2913 |
| 2022 | 264 | 544 | 534 | 175 | 511 | 366 | 113 | 7 | 101 | 2615 |
| Total | 1188 | 2011 | 2105 | 802 | 1909 | 1750 | 798 | 138 | 561 | 11262 |
