# Supplementary material for: The Prescription trends and dosing appropriateness analysis of novel oral anticoagulants in ischemic stroke patients: a retrospective study of 9 cities in China
Source: Front Pharmacol. 2024 Mar 12;15:1304139. doi: 10.3389/fphar.2024.1304139 (PMC10963614; doi:10.3389/fphar.2024.1304139)
Supplement: Supplementary file 6 [file Table2.docx]

**Table S2.** The number of prescriptions for NOACs from 2016 to 2022 [n (%) *].

| Year | Dabigatran | Rivaroxaban | Apixaban | Total NOACs | Total stroke prescriptions |
| --- | --- | --- | --- | --- | --- |
| 2016 | 485 (0.01) | 1339 (0.04) | 4 (0.00) | 1828 (0.05) | 3589160 |
| 2017 | 1241 (0.03) | 2026 (0.05) | 1 (0.00) | 3268 (0.08) | 3900698 |
| 2018 | 2573 (0.06) | 4876 (0.12) | 8 (0.00) | 7457 (0.19) | 4009439 |
| 2019 | 3603 (0.09) | 7541 (0.18) | 6 (0.00) | 11150 (0.26) | 4211022 |
| 2020 | 3052 (0.09) | 8094 (0.23) | 1 (0.00) | 11147 (0.32) | 3451189 |
| 2021 | 3309 (0.08) | 10631 (0.26) | 58 (0.00) | 13998 (0.35) | 4019602 |
| 2022 | 2339 (0.07) | 10746 (0.30) | 81 (0.00) | 13166 (0.37) | 3573514 |

Note: * Represents the percentage of NOACs prescriptions in total stroke prescriptions per year.
