# Supplementary material for: The Prescription trends and dosing appropriateness analysis of novel oral anticoagulants in ischemic stroke patients: a retrospective study of 9 cities in China
Source: Front Pharmacol. 2024 Mar 12;15:1304139. doi: 10.3389/fphar.2024.1304139 (PMC10963614; doi:10.3389/fphar.2024.1304139)
Supplement: Supplementary file 7 [file Table5.DOCX]

**Table S5**. Annual drug costs of apixaban in different cities.

| **Year** | **Beijing** | **Chengdu** | **Guangzhou** | **Harbin** | **Hangzhou** | **Shanghai** | **Shenyang** | **Tianjin** | **Zhengzhou** | **Total drug cost/CNY** |
| --- | --- | --- | --- | --- | --- | --- | --- | --- | --- | --- |
| 2016 | 0.00 | 0.00 | 172.28 | 0.00 | 0.00 | 0.00 | 0.00 | 0.00 | 0.00 | 172.28 |
| 2017 | 0.00 | 0.00 | 89.78 | 0.00 | 0.00 | 0.00 | 0.00 | 0.00 | 0.00 | 89.78 |
| 2018 | 0.00 | 0.00 | 395.30 | 0.00 | 1519.98 | 0.00 | 78.98 | 0.00 | 0.00 | 1994.26 |
| 2019 | 0.00 | 0.00 | 260.01 | 0.00 | 6079.92 | 0.00 | 0.00 | 0.00 | 0.00 | 6339.93 |
| 2020 | 0.00 | 0.00 | 796.74 | 0.00 | 1713.32 | 0.00 | 0.00 | 0.00 | 0.00 | 2510.06 |
| 2021 | 0.00 | 792.96 | 1072.12 | 0.00 | 439.04 | 1295.64 | 0.00 | 0.00 | 554.68 | 4154.44 |
| 2022 | 0.00 | 4750.68 | 717.36 | 0.00 | 374.36 | 1111.56 | 297.36 | 0.00 | 237.72 | 7489.04 |

Note: CNY, Chinese yuan.
