# Supplementary material for: The Prescription trends and dosing appropriateness analysis of novel oral anticoagulants in ischemic stroke patients: a retrospective study of 9 cities in China
Source: Front Pharmacol. 2024 Mar 12;15:1304139. doi: 10.3389/fphar.2024.1304139 (PMC10963614; doi:10.3389/fphar.2024.1304139)
Supplement: Supplementary file 8 [file Table3.DOCX]

**Table S3**. Annual drug costs of dabigatran in different cities.

| **Year** | **Beijing** | **Chengdu** | **Guangzhou** | **Harbin** | **Hangzhou** | **Shanghai** | **Shenyang** | **Tianjin** | **Zhengzhou** | **Total drug cost/CNY** |
| --- | --- | --- | --- | --- | --- | --- | --- | --- | --- | --- |
| 2016 | 42655.80 | 534.60 | 35652.68 | 9471.00 | 71757.00 | 40919.51 | 34202.02 | 4653.30 | 10216.80 | 250062.71 |
| 2017 | 59730.80 | 0.00 | 111784.54 | 3352.00 | 169899.30 | 98674.35 | 88943.91 | 5527.62 | 5889.00 | 543801.52 |
| 2018 | 157800.44 | 26027.70 | 145267.92 | 10046.04 | 471061.38 | 129495.10 | 108602.60 | 7321.24 | 24562.10 | 1080184.52 |
| 2019 | 320697.64 | 85499.82 | 209999.72 | 39772.76 | 445617.58 | 139265.36 | 65819.36 | 7244.78 | 47177.68 | 1361094.70 |
| 2020 | 396471.00 | 108330.24 | 193894.63 | 25262.24 | 449325.97 | 156450.72 | 43433.56 | 4922.48 | 30636.55 | 1408727.39 |
| 2021 | 329030.35 | 119973.24 | 171964.44 | 27134.66 | 341192.46 | 177317.86 | 40804.40 | 4703.53 | 33900.40 | 1246021.34 |
| 2022 | 136457.90 | 101830.89 | 56640.67 | 15021.57 | 154590.54 | 35271.92 | 12648.91 | 349.53 | 8436.18 | 521248.11 |

Note: CNY, Chinese yuan.
