# Supplementary material for: The Prescription trends and dosing appropriateness analysis of novel oral anticoagulants in ischemic stroke patients: a retrospective study of 9 cities in China
Source: Front Pharmacol. 2024 Mar 12;15:1304139. doi: 10.3389/fphar.2024.1304139 (PMC10963614; doi:10.3389/fphar.2024.1304139)
Supplement: Supplementary file 9 [file Table4.DOCX]

**Table S4**. Annual drug costs of rivaroxaban in different cities.

| **Year** | **Beijing** | **Chengdu** | **Guangzhou** | **Harbin** | **Hangzhou** | **Shanghai** | **Shenyang** | **Tianjin** | **Zhengzhou** | **Total drug cost/CNY** |
| --- | --- | --- | --- | --- | --- | --- | --- | --- | --- | --- |
| 2016 | 64839.57 | 12842.37 | 93316.43 | 38981.17 | 104623.11 | 22372.88 | 97121.49 | 10586.80 | 9622.40 | 454306.22 |
| 2017 | 85369.48 | 56341.10 | 119519.33 | 77988.05 | 93029.94 | 40285.37 | 107448.54 | 36516.22 | 4984.80 | 621482.83 |
| 2018 | 237997.40 | 234147.64 | 168148.80 | 47986.20 | 150282.00 | 109807.64 | 136715.64 | 58174.40 | 27569.00 | 1170828.72 |
| 2019 | 280867.92 | 236479.24 | 242877.65 | 85327.00 | 437956.80 | 221716.80 | 187470.72 | 94201.40 | 65502.84 | 1852400.37 |
| 2020 | 355541.72 | 303988.88 | 258574.64 | 91698.04 | 689310.00 | 321158.68 | 169896.24 | 132635.60 | 95666.68 | 2418470.48 |
| 2021 | 489331.15 | 327217.57 | 304794.79 | 150825.16 | 722360.08 | 438429.42 | 191187.14 | 168215.26 | 86112.77 | 2878473.34 |
| 2022 | 320233.85 | 129267.80 | 56350.02 | 58998.06 | 360877.44 | 105348.42 | 41719.62 | 44712.18 | 33864.72 | 1151372.11 |

Note: CNY, Chinese yuan.
