# Supplementary material for: The Prescription trends and dosing appropriateness analysis of novel oral anticoagulants in ischemic stroke patients: a retrospective study of 9 cities in China
Source: Front Pharmacol. 2024 Mar 12;15:1304139. doi: 10.3389/fphar.2024.1304139 (PMC10963614; doi:10.3389/fphar.2024.1304139)
Supplement: Supplementary file 10 [file Table6.docx]

**Table S6**. The number of appropriate and inappropriate dosing prescriptions from 2016 to 2022.

| Year | Total number of prescriptions | Appropriate dosing prescriptions, n (%) | Inappropriate dosing prescriptions, n (%) |
| --- | --- | --- | --- |
| 2016 | 1755 | 1569 (89.40) | 186 (10.60) |
| 2017 | 3127 | 2745 (87.78) | 382 (12.22) |
| 2018 | 7167 | 6103 (85.15) | 1064 (14.85) |
| 2019 | 10745 | 8894 (82.77) | 1851 (17.23) |
| 2020 | 10798 | 8547 (79.15) | 2251 (20.85) |
| 2021 | 13501 | 10588 (78.42) | 2913 (21.58) |
| 2022 | 12490 | 9875 (79.06) | 2615 (20.94) |
