# Supplementary material for: The Prescription trends and dosing appropriateness analysis of novel oral anticoagulants in ischemic stroke patients: a retrospective study of 9 cities in China
Source: Front Pharmacol. 2024 Mar 12;15:1304139. doi: 10.3389/fphar.2024.1304139 (PMC10963614; doi:10.3389/fphar.2024.1304139)
Supplement: Supplementary file 11 [file Table10.docx]

**Table S10**. The number of appropriate and inappropriate dosing prescriptions in different types of cities from 2016 to 2022.

| Year | Ap in first-tier cities | IAp in first-tier cities | Tp in first-tier cities | Ap in other cities | IAp in other cities | Tp in other cities |
| --- | --- | --- | --- | --- | --- | --- |
| 2016 | 761 | 43 | 804 | 808 | 143 | 951 |
| 2017 | 1613 | 189 | 1802 | 1132 | 193 | 1325 |
| 2018 | 3165 | 420 | 3585 | 2938 | 644 | 3582 |
| 2019 | 4588 | 700 | 5288 | 4306 | 1151 | 5457 |
| 2020 | 4380 | 939 | 5319 | 4167 | 1312 | 5479 |
| 2021 | 5905 | 1588 | 7493 | 4683 | 1325 | 6008 |
| 2022 | 4833 | 1164 | 5997 | 5042 | 1451 | 6493 |

Note: Ap, appropriate dosing prescriptions; IAp, inappropriate dosing prescriptions; Tp, total prescriptions. First-tier cities include Beijing, Guangzhou and Shanghai. Other cities include Chengdu, Harbin, Hangzhou, Shenyang, Tianjin and Zhengzhou.
